# Malarial Haematuria

**Published:** 1898-04

**Authors:** R. L. Denman

**Affiliations:** Durst, Texas


For the Texas Medical Journal.

### Malarial Haematuria.

BY R. L. DENMAN, M. D., DURST, TEXAS.

Drs. Briggs and Brodnax, in their papers on malaria, assert that quinine will not only not cure malaria—in face of the accumulated experience of thousands of intelligent observers to the contrary—but that it will produce hæmaturia. This position I cannot endorse. I do not believe that quinine is ever the cause of hæmaturia, or that it has ever "helped to send our patients to the better world."

Hæmaturia is not a disease, but a diseased condition, the result of a poison in the system; a specific organism—*plasmodium malariæ*—which has the effect of producing an engorgement or congestion of the kidneys, and hemorrhage is a result of that engorgement. The hemorrhage is, in these cases, as occasionally in other conditions, critical, not infrequently salutary, and we see the patient immediately get better, if the case is of not too long standing or the hemorrhage excessive. The condition that results in the hemorrhage is described in the works on the subject as "*Renal Malarial Hæmorrhagica.*" It is called by the people "black jaundice," because of the color of the discharges and the icteroid appearance generally, of the patient. This name I suppose was first given it by some "doctor" who had no knowledge of its pathological characteristics.

I have carefully studied now nineteen cases of malarial hæmaturia, and I will endeavor to give briefly my views upon the disease, and my conclusions. My studies of this disease extended over a period of three years.

Every case seen by me was ushered in by an initial chill, with pernicious symptoms, resembling the old time so-called "congestive chill" (all chills are congestive; it is only a matter of degree of congestion). It was most apt to occur in cases of malaria which had been neglected, or not properly treated in the beginning, and never as a result of quinine, but *because quinine had not been given* at the time when it, and it alone, could have arrested the progress of the malarial invasion! I have seen cases of hæmaturia which, it had been alleged, were produced by quinine; but we could with as much propriety say that it had been due to overloaded stomach and bowels, or to a suppression of the skin's action; want of elimination by that route. These cases had not created any suspicion on the part of the patient. They had not been aware that they were sick until bloody urine was being passed; many cases do not stop work.

The history in all the nineteen cases I had under observation during the time mentioned, showed that they had been chilling for some time, and presented what I regard as the characteristic conditions of malarial toxæmia—complexion pale and muddy, pallor, with enlargement of spleen and liver; a hyperplasia of tissue, generally called malarial cachexia, the effects of the malarial poison. Nor is the bloody urine the result of hyperpyrexia. I have seen it occur when the temperature was not over 103, especially if the cold stage has been prolonged. There is more or less cyanosis present, the pulse thready and rapid, and disproportionate to the temperature. The greater number of my cases, however, did present a higher temperature—106° and even 108°. These cases pass bloody urine in a short time after seizure. They complain of severe lumbar pain, and present an anxious countenance—from fright, I believe--and as this adds to the gravity of the case the physician should endeavor to calm the fears and excite hope and confidence.

As to the pathology of hæmaturia it is needless to speak, as you will find it in all the latest text books. I think the jaundice is caused by some chemical metabolism in the damaged and altered blood and the waste products which have not been eliminated by the proper channels, but are retained in the circulation; and not by the inhibition of bile as is held by some. It is a vicarious action, renal action being arrested. This is the patient's only salvation; the poisonous matter, chiefly urea, must find vent in some way or death will ensue. Hence, where there has been much hemorrhage from the kidneys there is usually suppression of urine; and then the jaundiced discoloration of the skin, and the vomited debris, appear. I had only one exception to this in my nineteen cases. In this one case there was not suppression of urine, although the urine voided by this patient was so bloody that it clotted upon being allowed to stand. The temperature of this case was 108°, and the pulse almost too fast to count. In ten hours, under proper treatment, the urine cleared up. He had had two chills, with a malignant tendency. I attribute to the heavy doses of quinine given him the prevention of suppression, and consequent uremia, the sedative effect of the quinine in large doses. It was administered while yet he was in the cold stage. Nevertheless, the jaundice in this case was as marked as in those in which uremia occurred. It must have been due to damaged—partly disorganized—blood, the effect of the poison, or to chemical changes not explainable. I do not think that quinine can be held responsible for the hemorrhage or any of the resultant damage; on the contrary, where there has been shown a marked tendency to hæmaturia—dark colored urine following the last chill before I administered the quinine, which I gave in full doses—the quinine has appeared to me to arrest or prevent it, as I would find after the first dose or so the urine cleared up and the pains relieved—those distressing lumbar pains which precede usually a hemorrhage from kidneys. The chill which I believe would have occurred next day was prevented and the impending trouble was averted.

The prognosis depends upon the amount of damage which has been done to kidneys, blood and the nervous system, as well as on prompt and proper treatment. There is much in knowing when to give quinine and when not to give it. My plan is, if the patient's urine is very bloody and there are uremic symptoms, recognized by copious watery dark vomited matter, and icterus rapidly making its appearance, with more or less incoherent talk, or a somnolent condition, I do not give the drug. I have observed that the uremia soon mitigates the malarial trouble; at least I so infer, because, after the appearance of the uremic symptoms I have never seen a patient have a chill; and the fever soon gradually declines; but I have given quinine in the beginning of some cases, and seen, in a few hours, that it was contraindicated; that is, in the doses usually given—sedative. I do not think that small doses would do any harm; but would it do any good? There are other remedies which I use instead, such as stimulants which meet the demands. Unless, as I said, the case shows a damaged state of the blood and kidneys sufficient to cause uremia, I administer quinine and push it to cinchonism as rapidly as I can, to insure keeping off next chill; it matters not how much blood may be passing by the kidneys. When the quinine is pushed thus, by the time the next chill is due the urine has usually cleared up and excepting a little prostration the patient is not much the worse for the attack. This is of course to be met by appropriate measures. As adjunct to the main remedy I stimulate the various emunctories assisting them in the task of elimination of debris. I usually give ten grains of calomel to an adult, combined with synergistics, and divided into four doses, one every hour, insuring free action, if necessary, by sulph. magnesia afterwards. I usually give hypodermically in appropriate cases, nitroglycerine gr. $\frac{1}{100}$, strychnia nit. gr. $\frac{1}{60}$, and pilocarpine gr. $\frac{1}{10}$, every four to six hours as needed. In from ten to thirty-six hours the bowels will have been thoroughly evacuated, the stomach quieted, the urine cleared up and the temperature will have fallen to normal. I allow cold water *ad libitum* in case of irritability of the stomach during the fever; if there is disposition to vomit, but nothing to vomit, warm water answers better in certain cases; mustard to the epigastrium, etc.

It is unwise in the extreme, in my opinion, to administer astringents with a view to stopping renal hemorrhage, as is the practice with some.

My observation has been that the strychnia and nitro-glycerine are the sheet anchor of safety, and will do more good in this condition than all the hemostatics in the shops. It comes nearer covering the indications as revealed by the pathology than any remedy I know. If the kidneys do not respond in a reasonable time I administer mild or even stimulating diuretics, allowing plenty of water meantime; I prefer turpentine in small doses, with other adjuncts, etc.

When convalescence has begun nitro-muriatic acid with mur.

tinct. iron, twenty drops every six hours, nourishing food, a little out-door exercise, stopping short of fatigue, etc.

A case of this kind requires the physician's constant and conscientious attention. Don't do too much, but meet all rational requirements, and do not leave your patient until he is out of danger.

By following the plan as above detailed, with more or less modification, I was enabled to cure seventeen of the nineteen cases I have referred to as constituting the subject of this incomplete paper. There is no use in relating the causes why I did not cure them all. Of course, any one can make excuses.

While there is no doubt that quinine has been abused, has been administered at the wrong time, in improper conditions of the system and in inappropriate doses, still it should not for that reason be tabooed and discarded. And the doctrine that quinine will cause hematuria is pernicious, if it should lead one to discard altogether what I regard as the sheet anchor of treatment in this dreaded and too frequently fatal malady. Convalescents should be sent for a short period to a pure atmosphere, out of the malarial section, and a good tonic may be taken with benefit. I prescribe:

℞ Quin. Bisulph............................................gr. c

Nitro muriatic acid dil....................ʒ v

Mur. Tr. iron..............................................ʒ v

Aqua destil. ad.........................℥ iv. m

Sig: Teaspoonful dose in water before meals.

I usually give Fowlers' solution at same time, after meals.

